# Crystal Structure, Chemical Bond, and Microwave Dielectric Properties of Ba_1−*x*_Sr*_x_*(Zn_1/3_Nb_2/3_)O_3_ Solid Solution Ceramics

**DOI:** 10.3390/molecules28083451

**Published:** 2023-04-13

**Authors:** Lei Xiao, Lianwen Deng, Yu Zhang, Ping Wu, Wenfei Zeng, Sen Peng

**Affiliations:** 1 Provincial Key Laboratory of Informational Service for Rural Area of Southwestern Hunan, Shaoyang University, Shaoyang 422000, China; 2 School of Physics and Electronics, Central South University, Changsha 410083, China

**Keywords:** Ba_1−*x*_Sr*_x_*(Zn_1/3_Nb_2/3_)O_3_, solid solution ceramics, microwave dielectric properties

## Abstract

Ba_1−*x*_Sr*_x_*(Zn_1/3_Nb_2/3_)O_3_ (BSZN) perovskite ceramics are prepared using the traditional solid-state reaction method. X-ray diffraction (XRD), Scanning electron microscopy (SEM), Raman spectroscopy, and X-ray photoelectron spectroscopy (XPS) were used to analyze the phase composition, crystal structure, and chemical states of BSZN ceramics, respectively. In addition, the dielectric polarizability, octahedral distortion, complex chemical bond theory, and PVL theory were investigated in detail. Systematic research showed that Sr^2+^ addition could considerably optimize the microwave dielectric properties of BSZN ceramics. The change in τ*_f_* value in the negative direction was attributed to oxygen octahedral distortion and bond energy (*E_b_*), and the optimal value of 1.26 ppm/°C was obtained at *x* = 0.2. The ionic polarizability and density played a decisive role in the dielectric constant, achieving a maximum of 45.25 for the sample with *x* = 0.2. The full width at half-maximum (FWHM) and lattice energy (U*_b_*) jointly contributed to improving the *Q* × *f* value, and a higher *Q* × *f* value corresponded to a smaller FWHM value and a larger U*_b_* value. Finally, excellent microwave dielectric properties (ε_r_ = 45.25, *Q* × *f* = 72,704 GHz, and τ*_f_* = 1.26 ppm/°C) were obtained for Ba_0.8_Sr_0.2_(Zn_1/3_Nb_2/3_)O_3_ ceramics sintered at 1500 °C for 4 h.

## 1. Introduction

In recent years, communication technologies such as satellite communication, mobile communication, and wireless local area networks have developed rapidly [1,2,3]. Media resonator-type filters have dominated the development of mobile communication systems. Microwave communication technology has been swiftly developed in dielectric resonator filters and numerous communication fields due to their characteristics [4,5,6,7]. Accordingly, the study of high-performance and stable microwave dielectric materials has become a current task. Microwave dielectric ceramics are widely used as indispensable electronic components because of their high dielectric constant (ε_r_), high-quality factor (*Q* × *f*), and low-temperature coefficient of resonant frequency (τ*_f_*) [8,9,10,11].

Microwave dielectric ceramics with the general formula of A(B′_1/3_B″_2/3_)O_3_ (A = Ba, Sr, Ca; B′ = Zn, Mg, Mn, Fe, Co, Ni, Cu; B″ = Nb or Ta) have been widely studied for their excellent microwave properties [9,12,13]. In recent years, in order to further promote the microwave dielectric properties and reduce the densification sintering temperature of ceramics, ion substitution and various additives were investigated in large-scale applications [14,15,16,17,18,19,20]. Especially in practical applications, it is important that the τ*_f_* value should be adjusted to near zero. According to previous studies, ceramics with perovskite structures often show good microwave dielectric properties [21,22,23,24]. Among these materials, Ba(Zn_1/3_Nb_2/3_)O_3_ (BZN) ceramics have perovskite structure and good microwave dielectric properties of ε_r_ = 41, *Q × f* = 54,000 GHz and τ*_f_* = 30 ppm/°C) [1]. BZN ceramics have been widely studied because of their lower dielectric loss and higher quality factor [13,15]. However, it cannot be put into practical application production because of the unacceptably high τ*_f_* value. Adding different dopants to obtain BZN ceramics with a near-zero τ*_f_* value is the focus of current research. Some studies on doping modification, such as A-site or B-site substitution for BZN ceramics, as shown below. The 0.95Ba (Zn_1/3_Nb_2/3_)O_3_ + 0.05BaZrO_3_ ceramics were studied and obtained good dielectric properties of ε_r_ = 42, *Q × f* = 96,000 GHz, and τ*_f_* = 27 ppm/°C [25]. As reported by Yue [26], Ba[(Zn_1−*x*_Co*_x_*)_1/3_Nb_2/3_]O_3_ (*x* = 0.8) ceramics could be well sintered at 1450 °C, and microwave dielectric properties of ε_r_ = 33, *Q × f* = 20,248 GHz and τ*_f_* = −0.11 ppm/°C were achieved. Although the replacement of Co^2+^ to Zn^2+^ lowered the τ*_f_* value to near zero, the *Q × f* value was seriously damaged. It was reported that Mg^2+^ substitution was used to improve the dielectric properties of BZN ceramics, yielding optimal dielectric properties of ε_r_ = 36, *Q × f* = 94,400 GHz, and τ*_f_* = 28.6 ppm/°C for Ba(Zn_1−*x*_Mg*_x_*)_1/3_Nb_2/3_)O_3_ ceramics at *x* = 0.4 [27]. Different proportions of La^3+^ and Ba^2+^ at the A-site in BZN were used to form solid solutions and obtained excellent dielectric properties of ε_r_ = 34, *Q × f* = 63,159 GHz and τ*_f_* = 5.21 ppm/°C [13].

The above results indicated that B-site ion substitution could not simultaneously achieve near-zero τ*_f_* value and high *Q × f* value for the BZN ceramics system; however, the A-site ion substitution could not only optimize the *Q × f* value but also tune the τ*_f_* value to near zero. Therefore, this work focuses on improving dielectric properties of BZN ceramics by replacing Ba^2+^ with Sr^2+^ at A-site. Unlike BZN with cubic structure, Sr(Zn_1/3_Nb_2/3_)O_3_ (SZN) is generally regarded as a perovskite hexagonal ordered structure, whose microwave dielectric performance is generally manifested as ε_r_ = 36.8, *Q* × *f* = 36,800 GHz, τ*_f_* = −25.2 ppm/°C [6]. After extensive investigation, Sr^2+^ doping will be used in this work to improve the microwave dielectric properties of BZN ceramics.

In this work, we hope that replacing Ba^2+^ with Sr^2+^ in different proportions can not only improve the dielectric properties of the ceramic system but also adjust the τ*_f_* value to near zero. By controlling the sintering temperature, the Ba_1−*x*_Sr*_x_*(Zn_1/3_Nb_2/3_)O_3_ ceramics were synthesized using the high-temperature solid phase method. The effects of Sr^2+^ concentrations on the microwave dielectric properties and crystal structure of BZN ceramics were investigated in detail. The relationship between the crystal structure and dielectric properties was obtained by employing Rietveld refinement.

## 2. Results and Discussion

**Figure 1:**
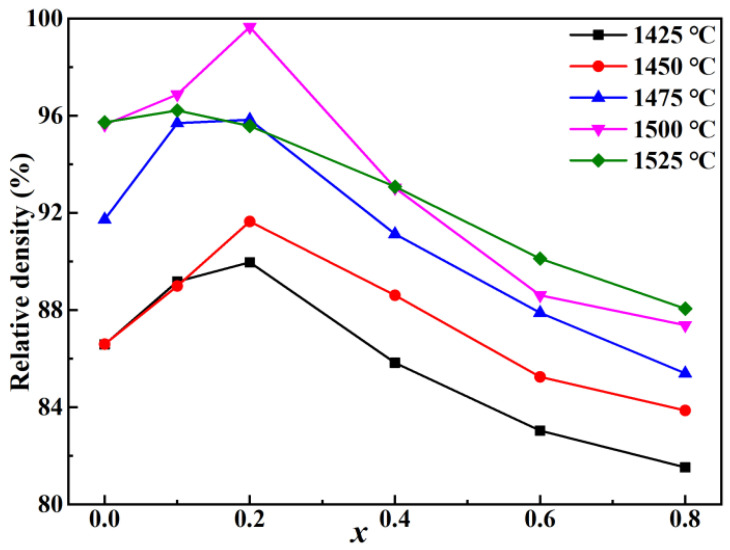
Relative density of Ba*_x_*Sr_1−*x*_(Zn_1/3_Nb_2/3_)O_3_ (*x* = 0–0.8) ceramics sintered at different temperatures.

Microwave dielectric properties of sintered ceramics are greatly affected by the relative density, which is related to the sintering temperature [6,9]. The relative densities of BSZN ceramics with different Sr^2+^ concentrations were obtained by sintering at 1425–1525 °C for 4 h, as shown in Figure 1. The relative density of the samples showed an overall increasing trend with the increasing sintering temperature. Nevertheless, the samples sintered at 1525 °C did not follow this trend, which was due to the decreased density caused by the evaporation of Zn^2+^ at higher temperatures [17]. For all samples sintered at 1425–1500 °C, it could be observed that the relative density firstly increased to the peak value at *x* = 0.2 and then decreased sharply with the increase in Sr^2+^ concentration. The optimum relative density of 99.7% was achieved at 1500 °C. Therefore, the samples sintered at 1500 °C are selected as the research object in this work.

**Figure 2:**
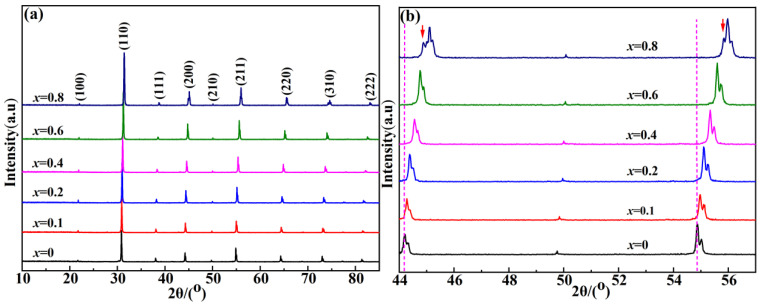
(**a**) XRD patterns of BSZN ceramics and (**b**) XRD patterns of BSZN ceramics in the 2θ range 44–57°.

Figure 2a displays the XRD patterns of the samples prepared in this work sintered at 1500 °C for 4 h. The obtained patterns were compared with the standard JCPDS card No. 39-1474. It was found that all phases could be matched, indicating that the sample obtained in this work was a pure phase, and there were no redundant secondary phases or impurities. Additionally, no reflection peak was observed in the low-angle reflection region of 10–20°. For all samples, the peaks of (200) and (211) had some deviations, as shown in Figure 2b. According to the Bragg equation, this phenomenon was due to the successful formation of a solid solution, and the diffraction peak shifted to a higher angle as the ionic radius of Sr^2+^ was smaller than that of Ba^2+^. The reflection peaks (44–57°/2θ) are amplified and analyzed in Figure 2b. It was found that the broadening and splitting of reflection peaks (200) and (211) were clearly observed between *x* = 0.6 and *x* = 0.8, as shown by the downward arrows in Figure 2b. The broadening and splitting of reflection peaks were due to the presence of 1:2 ordering with the increasing Sr^2+^ concentration. This phenomenon indicated the transformation of crystal structure from the disordered phase at the beginning to the pseudocubic phase, where disordered phases and ordered phases coexisted [6]. The underlying reason for the occurrence of the phase transition was the tilting of the oxygen octahedron [5]. Tilting of the oxygen octahedron enables the phase transition from the disordered phase to the ordered phase, that was, symmetry reduction.

**Figure 3:**
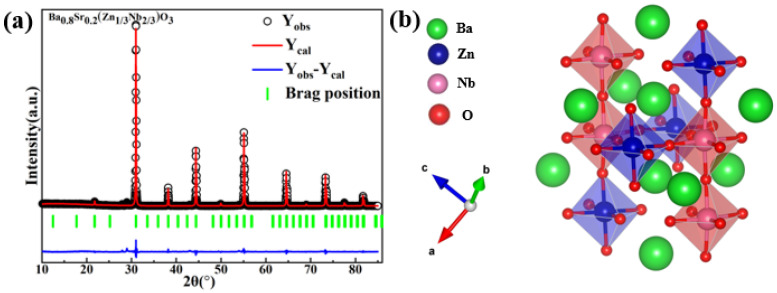
(**a**) The refinement plot of Ba_1−*x*_Sr*_x_*(Zn_1/3_Nb_2/3_)O_3_ (*x* = 0.2) ceramics (R_wp_ = 9.61%, R_p_ = 6.1%, and χ^2^ = 2.88); (**b**) The crystal structure patterns of Ba_1−*x*_Sr*_x_*(Zn_1/3_Nb_2/3_)O_3_ (*x* = 0.2) ceramics.

In order to better analyze the cubic structure of perovskite Ba_1−*x*_Sr*_x_*(Zn_1/3_Nb_2/3_)O_3_ ceramics, the atomic position corresponding to the Ba(Zn_1/3_Nb_2/3_)O_3_ with cubic structure and *Pm*$\overset{-}{3}$*m* space group was taken as the initial model. As shown in Figure 3a, the refinement data of Ba_1−*x*_Sr*_x_*(Zn_1/3_Nb_2/3_)O_3_ (*x* = 0.2) were obtained by the Rietveld refinement method using FullProf software [19], including observed and calculated data, peak locations, and differences. Additionally, the optimum R factor and fitting results (R_wp_ = 9.61%, R_p_ = 6.1%, R_exp_ = 5.66%, and χ^2^ = 2.88) were achieved, which indicated that the data obtained in this work were reliable.

Figure 3b presents the crystal structure of BSZN ceramics. The crystal diagram of BSZN ceramics with perovskite cubic structure was obtained by editing the CIF (Crystal Information File) using VESTA software [28]. The results showed that the metal ions were located in the center of the oxygen octahedron, each connected by shared vertices. With the increase in Sr^2+^ content, Ba^2+^ was replaced by Sr^2+^, and the crystal structure parameters were changed gradually.

The lattice parameters, unit cell volume, and bond angle were obtained by the Rietveld refinement. Table 1 displays the crystallographic data achieved from the Rietveld refinement of Ba_1−*x*_Sr*_x_*(Zn_1/3_Nb_2/3_)O_3_ ceramics. Careful examination of Table 1 showed that the lattice parameters (a, b, and c) and unit cell volume decreased monotonically with the increase in Sr^2+^ content, which was due to the difference in ionic radii between Sr^2+^ (1.44 Å) and Ba^2+^ (1.61 Å) [6]. The ionic radius of Sr^2+^ was smaller than that of Ba^2+^, resulting in the deterioration of lattice parameters and unit cell volume. In addition, combined with changes in the peaks (200) and (211) shown in the XRD pattern, the variations of the lattice parameters shown in Table 1 could be proven reasonable.

**Figure 4:**
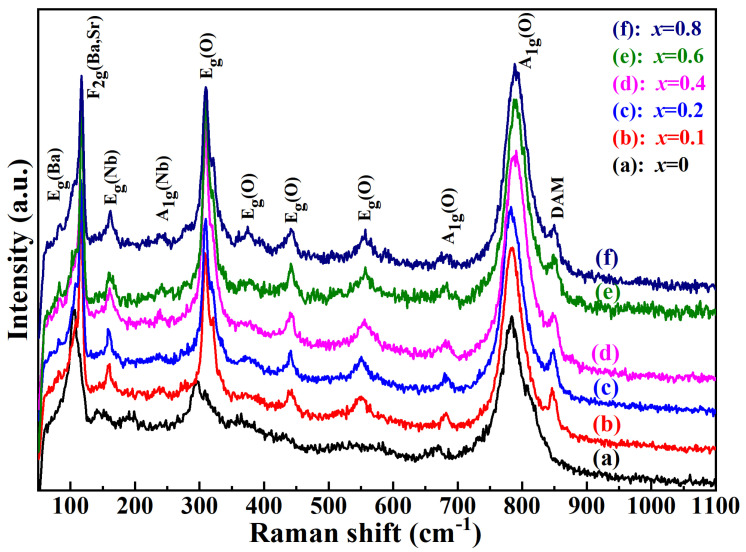
Raman spectra of Ba_1−*x*_Sr*_x_*(Zn_1/3_Nb_2/3_)O_3_ (*x* = 0–0.8) ceramics.

Figure 4 exhibits the Raman spectra of Ba_1−*x*_Sr*_x_*(Zn_1/3_Nb_2/3_)O_3_ (*x* = 0–0.8) ceramics sintering at 1500 °C for 4 h. Raman spectroscopy was sensitive to the subtle changes in crystal structure and cation substitution [2], thus it was used as a characterization technique to study the crystal structure of the samples. As shown in Figure 4, 4 active modes were observed, which were related to the spectra of the 1:1 ordered perovskites with *Fm*$\overset{-}{3}$*m* symmetry, and the position of the peaks shown in the Raman spectra were recorded separately in the (1) F_2g_(Ba, Sr) phonon modes around 105 cm^−1^, (2) the E_g_(O) phonon modes close to 380 cm^−1^, (3) the E_g_(O) phonon modes in the range of 430–450 cm^−1^, and (4) the A_1g_(O) phonon modes near 800 cm^−1^, corresponding to the stretch vibration of oxygen octahedron [22]. As shown in Figure 4, the F_2g_(Ba, Sr) modes (A_1g_ + E_g_), loaded at ~105 cm^−1^, correlated with a 1:1 ordered structure, which was attributed to the vibration of Ba and Sr atoms against the oxygen octahedron [2]. As shown in Figure 4, the peaks located at 150–350 cm^−1^ were associated with the 1:2 ordered phase [22]. The E_g_(Nb) modes near 171 cm^−1^ and 263 cm^−1^ originated from the vibration of Nb atoms, and the E_g_(O) modes near 300 cm^−1^, 376 cm^−1^, and 548 cm^−1^ arose from the twisting vibration of oxygen octahedrons along different directions. Additionally, the A_1g_(O) mode near 788 cm^−1^ was assigned as the stretching breath vibration of oxygen octahedrons along the c-axis, which could be used as an important identification method for different types of B-site ordering [2]. It was reported that the peak near 680 cm^−1^ was also regarded as A_1g_(O) mode, which was closely related to the localized 1:1 ordering [16,19,21]. Ultimately, the peak located at nearly 846 cm^−1^ was considered a defect-activated mode (DAM). It was reported that this mode was an active mode around the A_1g_(O) mode, which might be correlated with micro-defects such as complex boundaries [2]. Raman phonon mode was not only related to the crystal structure but also sensitive to the dielectric properties of sintered ceramics. The relationship between the FWHM of A_1g_(O) and *Q* × *f* value with Sr^2+^ concentration is shown in Figure 10.

**Figure 5:**
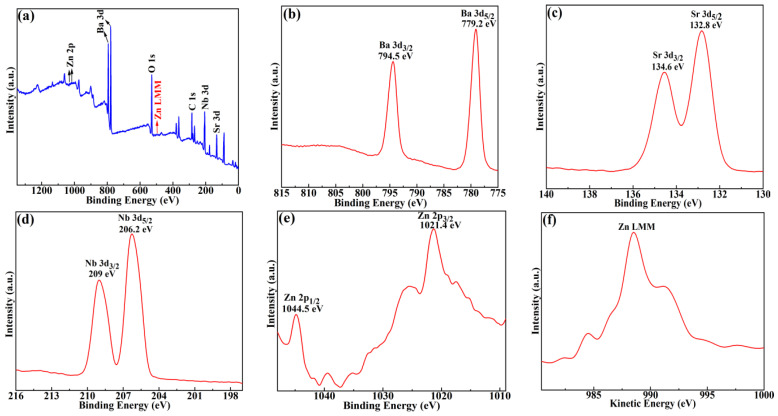
XPS spectra of Ba_1−*x*_Sr*_x_*(Zn_1/3_Nb_2/3_)O_3_ (*x* = 0.2) ceramics with (**a**) full spectra, (**b**) Ba 3d, (**c**) Sr 3d, (**d**) Nb 3d, (**e**) Zn 2p, and (**f**) Zn LMM.

**Figure 6:**
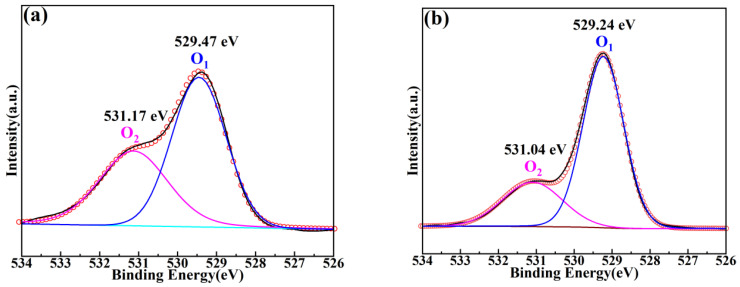
XPS spectra of O 1s for (**a**) undoped sample and (**b**) the sample with *x* = 0.2.

Figure 5 represents the XPS spectra of BSZN ceramics sintered at 1500 °C for 4 h. The presence of Ba 3d, Sr 3d, Zn 2p, Zn LMM, Nb 3d, O 1s, and C 1s in BSZN ceramics was determined by XPS analysis. The XPS data obtained in this work were all corrected for charge with reference to the C 1s peak of 284.8 eV. The narrow scan XPS spectrum of Ba 3d for BSZN ceramics was shown in Figure 5b, which consisted of spin-orbit doublet peaks Ba 3d_5/2_ and Ba 3d_3/2_ observed at 779.2 and 794.5 eV, respectively. Additionally, the difference between the 2 peaks after spin splitting was 15.3 eV, which was in good agreement with the characteristic spectrum of Ba^2+^ in XPS [24]. This phenomenon demonstrated the existence of Ba^2+^ in samples. The Sr 3d spectrum shown in Figure 5c displayed greatly separated spin-orbit 3d_5/2_ and 3d_3/2_ components (Δ = 1.4 eV) at 132.8 and 134.2 eV, corresponding to the characteristic spectrum of Sr with a valence of +2 [29]. The peaks of 3d_5/2_ and 3d_3/2_ of Nb for BSZN ceramics shown in Figure 5d were at 206.2 and 209 eV, respectively, with a difference of 2.8 eV, which was indexed to Nb^5+^ [30]. Unfortunately, the peak positions between Zn^0^ and Zn^2+^ tended to overlap, as shown in Figure 5e. So, Zn^2+^ was not possible to directly confirm from only the Zn 2p spectra. Therefore, it could be verified only by investigating the Zn LMM Auger spectrum, as shown in Figure 5f. The comparison of the Zn LMM Auger spectrum revealed the bigger chemical shifts of Zn^2+^ compared to Zn^0^, so the chemical state of Zn in BSZN ceramics could be assigned as Zn^2+^ [31]. The spectra of O 1s for an undoped sample and the sample with *x* = 0.2 were identified by peak fitting technique, and each of the O 1s could be divided into two peaks, as shown in Figure 6. The dominant peak (O_1_) for an undoped sample and the sample with *x* = 0.2 was loaded at 529.47 eV and 529.24 eV, respectively, assigned as O^2−^ [28]. Additionally, 2 shoulder peaks (O_2_) were all loaded at 531–533 eV, which was caused by the hydrocarbonates formation at the sample surface [32]. Moreover, with the incorporation of Sr^2+^, the peak positions of O_1_ and O_2_ all shifted to low binding energy, and the intensity ratios between O_2_ and O_1_ gradually decreased, with values of 13:20 and 9:20, respectively, which were caused by the replacement of Ba^2+^ with Sr^2+^ in Ba(Zn_1/3_Nb_2/3_)O_3_ ceramics. The above analysis demonstrated that the chemical states of Ba, Sr, Zn, Nb, and O were +2, +2, +2, +5, and −2, respectively.

**Figure 7:**
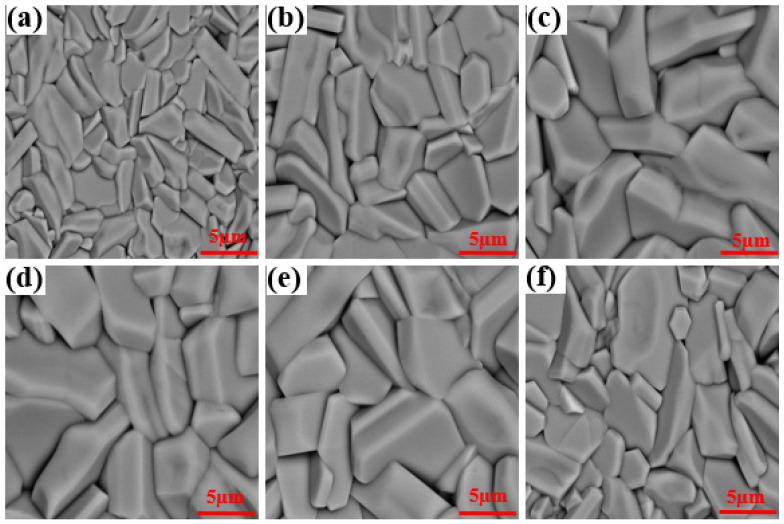
Backscatter SEM photographs of polished and annealed treatment fracture surfaces of Ba_1−*x*_Sr*_x_*(Zn_1/3_Nb_2/3_)O_3_ ceramics with (**a**) *x* = 0, (**b**) *x* = 0.1, (**c**) *x* = 0.2, (**d**) *x* = 0.4, (**e**) *x* = 0.6, and (**f**) *x* = 0.8.

**Figure 8:**
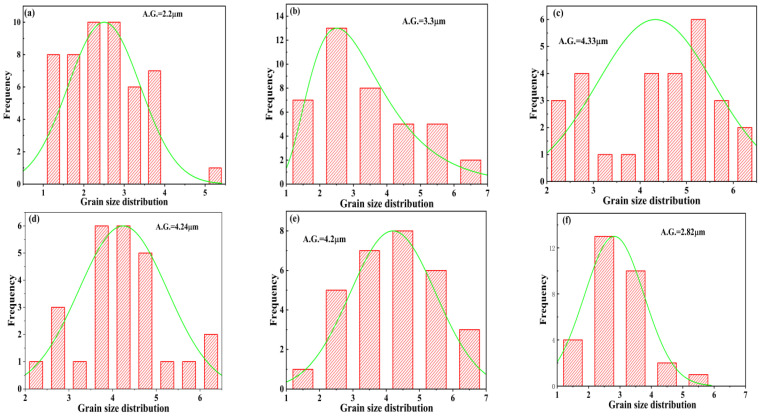
The average grain size of Ba_1−*x*_Sr*_x_*(Zn_1/3_Nb_2/3_)O_3_ ceramics with (**a**) *x* = 0, (**b**) *x* = 0.1, (**c**) *x* = 0.2, (**d**) *x* = 0.4, (**e**) *x* = 0.6, and (**f**) *x* = 0.8.

Figure 7 displays the Backscatter SEM photographs of polished and annealed treatment fracture surfaces of Ba_1−*x*_Sr*_x_*(Zn_1/3_Nb_2/3_)O_3_ ceramics sintered at 1500 °C for 4 h. The porosity and grain size were greatly affected by Sr^2+^ concentration. As *x* ≤ 0.2, with the increasing of Sr^2+^ concentration, the porosity gradually decreased, whereas the average grain size started to increase correspondingly, as presented in Figure 8, acquiring a maximum relative density of 99.7%. Notably, abnormal grains emerged at *x* ≥ 0.4, as displayed in Figure 7d–f. Abnormal grains could damage the microstructure of the samples, subsequently influencing the dielectric properties of the ceramic system [33].

Table 2 demonstrates the EDS analysis of the overall fracture surface for the BSZN ceramics with different Sr^2+^ concentrations. The EDS mapping and corresponding spectra for all samples are shown in Figures S1–S6. The results presented that the Sr atom ratio increased with Sr^2+^ concentration. The proportion of Zn deviated from the stoichiometric ratio in the chemical formula, as observed in Table 2, which was attributed to the volatilization of ZnO during the high-temperature sintering process [18]. As shown in Table 2, it could be seen that the sample with *x* = 0 presented a Ba/Nb elemental molar ratio of 26.71:17.59, close to that in Ba(Zn_1/3_Nb_2/3_)O_3_. For all samples except *x* = 0, the ratio of (Ba + Sr)/Nb was basically maintained at 1.5:1, corresponding to a combination of BZN and SZN, which also proved the formation of a solid solution.

**Figure 9:**
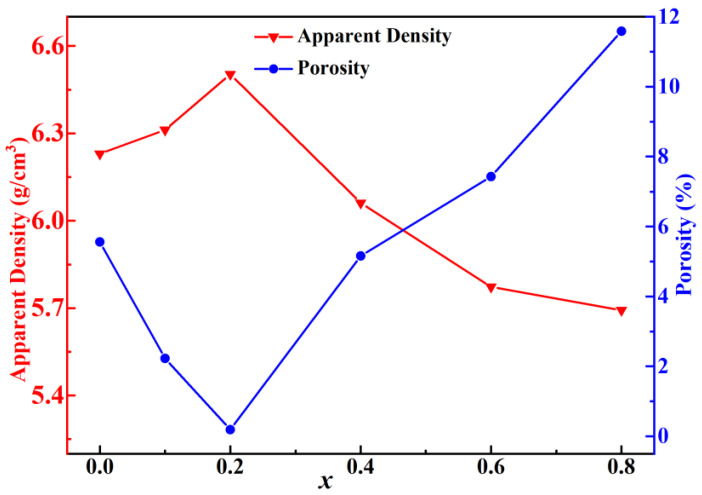
The variation of apparent density and porosity with *x* from 0 to 0.8.

Figure 9 reveals the relationship between the apparent density and porosity of the samples sintered at 1500 °C for 4 h. The overall apparent density showed a trend of first increasing and then decreasing with the increase in Sr^2+^ concentration. With the increasing of Sr^2+^ concentration, the apparent density firstly increased to the maximum value of 6.503 g/cm^3^ at *x* = 0.2, which was attributed to the successful formation of the solid solution by the substitution of Sr^2+^ for Ba^2+^ and the uniform grain growth shown in Figure 7c. At *x* > 0.2, the apparent density displayed a downward tendency accompanied by increasing porosity with Sr^2+^ concentration. Combined with Figure 7, this phenomenon was attributed to the increased pores caused by abnormal grain growth. The reaction-sintering manner was once more proved to obtain extraordinarily dense ceramic pellets.

The PVL theory of chemical bonding, developed by Phillips, Van Vechten, and Levine, is a dielectric description of chemical bonds in crystal structures. In this work, the PVL theory was used to split the crystal structure of Ba_1−*x*_Sr*_x_*(Zn_1/3_Nb_2/3_)O_3_ (*x* = 0–0.8) ceramics into the following bonding formulas, such as the *A_m_B_n_* form:

(1) $$\begin{array}{cc} {\mathrm{Ba}}_{3-3x}{\mathrm{Sr}}_{3x}{\mathrm{ZnNb}}_{2}O_{9} & =\;({\mathrm{Ba}}_{3-3x}{\mathrm{Sr}}_{3x}{)(1)}_{1/2}O{\left(1\right)}_{1}+({\mathrm{Ba}}_{3-3x}{\mathrm{Sr}}_{3x}{)(1)}_{1/2}O(2)+{\left({\mathrm{Zn}}_{1}{\mathrm{Nb}}_{2}\right)}_{1}O{\left(2\right)}_{1}+{\left({\mathrm{Nb}}_{1}{\mathrm{Zn}}_{2}\right)}_{1}O{\left(1\right)}_{1}+\\ & {\left({\mathrm{Nb}}_{1}{\mathrm{Zn}}_{2}\right)}_{1}O{\left(2\right)}_{1}+({\mathrm{Ba}}_{3-3x}{\mathrm{Sr}}_{3x}{)(2)}_{1/2}O{\left(1\right)}_{1}+({\mathrm{Ba}}_{3-3x}{\mathrm{Sr}}_{3x}{)(2)}_{3/2}O{\left(2\right)}_{3}, \end{array}$$

Generally speaking, the bond ionicity (*f_i_*) was closely related to the dielectric constant, and the specific relationship was as follows:

(2) $$\varepsilon_{r}=\frac{n^{2}-1}{1-f_{i}}+1,$$

where n was a refractive index that could be considered a constant. *f_i_* and ε_r_ were determined to be a positive relationship where ε_r_ increased with the increase in *f_i_*.

The calculation of PVL for the ionic and covalent properties of BSZN ceramics involved the following Equations (3)–(7) [34]:

(3) $$f_{i}^{\mu }=\frac{{\left(C^{\mu }\right)}^{2}}{{\left(E_{g}^{\mu }\right)}^{2}},$$

(4) $$f_{c}^{\mu }=\frac{{\left(E_{h}^{\mu }\right)}^{2}}{{\left(E_{g}^{\mu }\right)}^{2}},$$

(5) $${\left(E_{g}^{\mu }\right)}^{2}={\left(E_{h}^{\mu }\right)}^{2}+{\left(C^{\mu }\right)}^{2},$$

(6) $$E_{h}^{\mu }=39.74/{\left(d^{\mu }\right)}^{2.48},$$

(7) $$C^{\mu }=14.4b^{\mu }\exp(-\kappa_{s}^{\mu }r_{0}^{\mu })\times [\frac{m}{n}{\left(Z_{A}^{\mu }\right)}^{*}-{\left(Z_{B}^{\mu }\right)}^{*}]/r_{0}^{\mu },$$

$f_{i}^{\mu }$ and $f_{c}^{\mu }$ were defined as the ionic and covalent values of the μ bond, respectively. $E_{g}^{\mu }$ was interpreted as the average energy band, part of which was composed of isotropic polarization $E_{h}^{\mu }$ determined by the covalence of chemical bonds, where $d^{\mu }$ was defined as the length of the μ bond. The m and n were determined as the number of anions and cations in *A_m_B_n_*, respectively. $r_{0}^{\mu }$ was defined as the ionic radius of the μ bond. The heteropole part $C^{\mu }$ shown in Equation (7) was determined by the correction factor ($b^{\mu }$), the effective valence electron number ${\left(Z_{A}^{\mu }\right)}^{*}$, ${\left(Z_{B}^{\mu }\right)}^{*}$, and the Thomas–Fermi shielding factor $\exp(-\kappa_{s}^{\mu }r_{0}^{\mu })$ was determined according to the following Equations (8)–(10) [35]:

(8) $$\kappa_{s}^{\mu }={\left(\frac{4k_{F}^{\mu }}{\pi \alpha_{B}}\right)}^{\frac{1}{2}},$$

(9) $$({k_{F}^{\mu })}^{3}=3\pi^{2}({N_{e}^{\mu })}^{*},$$

(10) $$({N_{e}^{\mu })}^{*}=\frac{{\left(n_{\mu }\right)}^{*}}{v_{b}^{\mu },}$$

where $\alpha_{B}$ was the Bohr radius whose value was regarded as a constant of 0.5292 Å. $k_{F}^{\mu }$ was calculated from the *μ* bond ${\left(n_{\mu }\right)}^{*}$ and the bond volume ${(v}_{b}^{\mu }$), corresponding to Equations (11) and (12).

(11) $$({n_{\mu })}^{*}=\frac{{\left(Z_{A}\right)}^{*}}{N_{cA}^{\mu }}+\frac{{\left(Z_{B}\right)}^{*}}{N_{cB}^{\mu }},$$

(12) $$v_{b}^{\mu }=\frac{{\left(d^{\mu }\right)}^{3}}{\sum_{v}{{(d}^{\mu })}^{3}*N_{b}^{v}},$$

where $N_{cA}^{\mu }$, $N_{cB}^{\mu }$ were the coordination numbers of A and B atoms attached to *μ* bonds in a cell, respectively. $N_{b}^{v}$ was defined as the bond density of *μ*-bonds (number of *μ* bonds in 1 cm^3^), which could be obtained from the refined crystal structure. The bond energy (*E_b_*) was closely related to the τ*_f_* value of ceramic materials [36], whose definition was shown in Equations (13)–(18):

(13) $$E=\;\sum E_{b}^{\mu },$$

*E*_*b*_ = *t*_*c*_*E*_*c*_ + *t*_*i*_*E*_*i*_, (14)

1 = *t*_*i*_ + *t*_*c*_, (15)

(16) $$t_{i}=|\frac{{(S}_{A}-S_{B})/∆S_{B}}{2}|,$$

(17) $$E_{i}=\frac{33,200}{d^{\mu }},$$

(18) $$E_{c}=\frac{\left({r_{cA}+r}_{cB}\right)}{d^{\mu }}{\left(E_{A-A}E_{B-B}\right)}^{1/2},$$

where *E_A−A_* and *E_B−B_* were defined as homonuclear bond energies, *t_c_* and *t_i_* were covalent and ionic co-mixing factors, *S_A_* and *S_B_* represented the electronegativity of A and B ions, and Δ*S_B_* represented the change in electronegativity with a value of 3.01. *E_i_* and *E_c_* were determined as the energies contributed by the ionic and covalent bonds, respectively.

It is well known that the lattice energy (U*_b_*) was an intrinsic influence on the variation of the *Q* × *f* value, and it was defined as a concept representing the binding capacity between ions. The U*_b_* value could be calculated by the following Equations (19)–(21):

(19) $$U_{b}^{\mu }=U_{bc}^{\mu }+U_{bi}^{\mu },$$

(20) $$U_{bc}^{\mu }=2100m\frac{(Z_{+}^{\mu }{)}^{1.64}}{(d^{\mu }{)}^{0.75}}f_{c}^{\mu },$$

(21) $$U_{bi}^{\mu }=1270\frac{(m+n)Z_{+}^{\mu }Z_{-}^{\mu }}{d^{\mu }}\left(1-\frac{0.4}{d^{\mu }}\right)f_{i}^{\mu },$$

where $U_{bi}^{\mu }$ and $U_{bc}^{\mu }$ represented the lattice energy of the ionic part and the covalent part of the constituent U*_b_*, respectively. m and n were the parameters of the *A_m_B_n_* binary system. $Z_{+}^{\mu }$ and $Z_{-}^{\mu }$ represented the parameters of the cationic and anionic valence states, respectively.

Table 3 shows the ionic and covalent properties of the different bonds in the BZN ceramics sintered at 1500 °C for 4 h, which were obtained by the PVL theory. As shown in Table 3, the ionicity magnitudes in BZN ceramics revealed that *f_i_*(Ba-O) > *f_i_*(Nb-O) > *f_i_*(Zn-O), where the ionicity of Ba-O bond reached a maximum of 85.80%. Therefore, the substitution of Ba sites could effectively improve the microwave dielectric properties of Ba(Zn_1/3_Nb_2/3_)O_3_ ceramics, and the experimental results were shown in the following analysis.

It was reported that ε_r_ was largely influenced by the polarizability of the materials, density, and porosity [21,22]. In order to study the changing trend of ε_r_, the theoretical dielectric polarizability (α_theo_) reported by Shannon et al. was invoked as formulated in Equation (22) [37].

α_theo_ = α(Ba_1–*x*_Sr*_x_*(Zn_1/3_Nb_2/3_)O_3_) = *x*α(Sr^2+^) + (1 – *x*)α(Ba^2+^) + 1/3α(Zn^2+^) + 2/3α(Nb^5+^) + 3α(O^2−^), (22)

where α(Sr^2+^) = 4.24 Å^3^, α(Ba^2+^) = 6.40 Å^3^, α(Zn^2+^) = 2.04 Å^3^, α(Nb^5+^) = 3.97 Å^3^, α(O^2−^) = 2.01 Å^3^. On the basis of the Clausius–Mossotti equation [37], the relationship between ε_r_ and observed dielectric polarizability (α_obs_) was defined as follows:

(23) $$\alpha_{\mathrm{obs}}=\frac{V_{m}(\varepsilon_{r}-1)}{b^{\prime }(\varepsilon_{r}+2)},$$

where ε_r_, V_m_ and b′ were the measured dielectric constant, molar volume (V_cell_/Z) and a constant (4π/3), respectively. Furthermore, the deviation (Δ) between α_theo_ and α_obs_ of Ba_1–*x*_Sr*_x_*(Zn_1/3_Nb_2/3_)O_3_ obeyed

(24) $$\Delta =|\frac{\alpha_{obs}-\alpha_{theo}}{\alpha_{obs}}\times 100\%|.$$

The relationships of α_theo_, α_obs_, and ε_r_ are listed in Table 4. It could be directly observed from Table 4 that α_theo_ and α_obs_ showed the same decreasing trend. This phenomenon was caused by the difference between α(Sr^2+^) and α(Ba^2+^). However, ε_r_ firstly increased to the maximum value of 45.25 at *x* = 0.2 and then decreased. When *x* ≤ 0.2, the upward trend of ε_r_ was attributed to the fact that the higher density occupation of the sample determined the dominance of ε_r_. As the Sr^2+^ concentration further increased for *x* > 0.2, ε_r_ started to decrease with the decreasing of α_theo_ and α_obs_, which was primarily determined by ionic polarizability [1]. Careful examination of Table 4 showed that the variation of α_theo_ was in good agreement with that of α_obs_, which indicated that the deterioration of ε_r_ was mainly attributed to the lower ionic polarizability of Sr^2+^ compared with that of Ba^2+^. The formation of deviation (Δ) was mainly because α_theo_ and α_obs_ were derived from many semi-empirical equations of oxides and fluorides. Moreover, as shown in Table 4, the obtained deviations were so small that the results were reliable.

With the addition of Sr^2+^, the atomic interactions in Ba_1−*x*_Sr*_x_*(Zn_1/3_Nb_2/3_)O_3_ solid solutions were elongated or compressed inevitably, resulting in structural variations. The bond length of the oxygen octahedron was correlated with the lattice energy and bond iconicity. Moreover, the variation of oxygen octahedrons had a strong effect on the dielectric properties. Thus, the octahedral distortion of BSZN ceramics sintered at 1500 °C for 4 h was calculated using the octahedral distortion formula [4].

(25) $$\mathrm{Octahedral}\;\mathrm{distortion}\;(\Delta_{\mathrm{octahedral}})=\frac{1}{6}(\frac{R_{i}-\overset{-}{R}}{\overset{-}{R}}{)}^{2},$$

where R_i_ and $\overset{-}{R}$ were the individual bond length and average bond length of oxygen octahedrons, respectively.

V_ij_ was defined as the sum of all of the valences from a given atom i, and this was calculated in Equations (26) and (27) [38]:

(26) $$V_{\mathrm{ij}}=\sum v_{ij},$$

(27) $$v_{\mathrm{ij}}=\exp\left(\frac{R_{ij}-d_{ij}}{b^{\prime }}\right),$$

where R_ij_ represented the bond valence parameter, d_ij_ indicated the length of a bond between atoms i and j, and b’ was a universal constant with a value of 0.37 Å.

Table 5 records the variations of bond length, octahedral distortion, bond energy, and τ*_f_* value for Ba_1−*x*_Sr*_x_*(Zn_1/3_Nb_2/3_)O_3_ (*x* = 0–0.8) ceramics with the increasing Sr^2+^ concentration. As shown in Table 5, the variation range of the τ*_f_* value for all samples was 27.72~−15.72 ppm/°C, and the τ*_f_* value displayed a downward tendency with the increasing of Sr^2+^ concentration. Generally speaking, the τ*_f_* value depended very strongly heavily on the bond energy, the distortion of the oxygen octahedron and the bond valence changes in the constituent ions [39]. For one thing, *E_b_* was defined as the binding capacity of the bond between the cation and the anion, which was inversely related to the absolute value of τ*_f_*. This phenomenon was attributed to the fact that the crystal structure was highly dependent on the value of *E_b_*. With the increasing *E_b_* value, the crystal structure of the sample became more and more stable, and then the absolute value of τ*_f_* decreased. Similar changes in the two parameters shown in Table 5 also proved that the larger the *E_b_* value, the more stable the crystal structure. Simultaneously, the corresponding τ*_f_* was close to zero. For another, with the increase in B-site bond valence, the degree of tilting on oxygen octahedrons and the bond strength between B-site cation and oxygen increased. Consequently, the restoring force to the tilting recovers increased, thereby resulting in a decrease of τ*_f_* value. Therefore, the main reason for the change of τ*_f_* value from positive to negative was ascribed to the three factors: bond energy, distortion of the oxygen octahedron, and B-site bond valence. At last, a near-zero τ*_f_* value of 1.26 ppm/°C could be acquired for the sample with *x* = 0.2.

**Figure 10:**
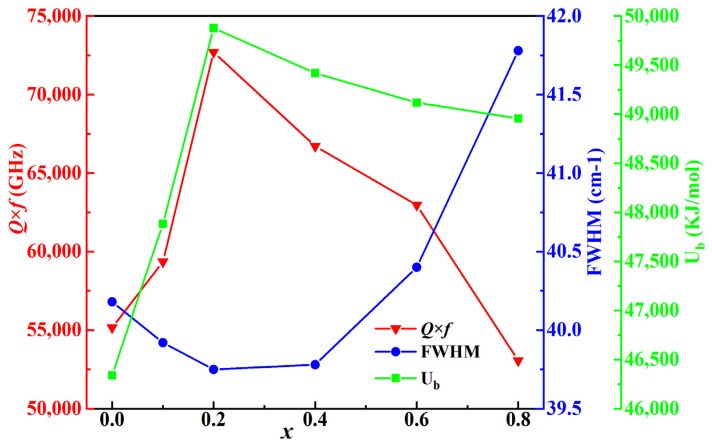
The *Q* × *f* value, FWHM and U*_b_* of BSZN ceramics.

Figure 10 represents the interrelationships between the *Q* × *f* values, FWHM of A_1g_(O) and U*_b_* for BSZN ceramics sintered at 1500 °C for 4 h. FWHM was considered a characterization technology to determine the ordering feature rather than the peak position or peak value. A smaller FWHM presented less interference between phonons and a longer lifetime, corresponding to the higher ordering of the samples [19,20]. According to the ref [2], the microwave dielectric properties of ceramics were closely related to FWHM, which was one of the factors affecting microwave dielectric properties. It was worth noting that the sample with *x* = 0.2 had the smallest FWHM of A_1g_(O) mode and the highest *Q* × *f* value, which was similar to the result of ref [40]. As shown in Figure 10, the measured *Q* × *f* values first increased from 55,158 GHz to 72,704 GHz and then gradually decreased, similar to the variation trend of U*_b_*. Therefore, U*_b_*, obtained by PVL theory calculation, was considered a key factor to determine the *Q* × *f* value, and *Q* × *f* showed the same changing trend as that of U*_b_*. It was noteworthy that with the increasing Sr^2+^ content, the A-site ions in the BSZN samples proved to be strongly affected by internal losses. It could be clearly observed that the total lattice energy of the BSZN sample increased from 46,344 KJ/mol to 49,877 KJ/mol when *x* increased from 0 to 0.2, which played a non-negligible effect on the enhancement of *Q × f*. According to XRD analysis, all ceramics were pure phase. Therefore, the *Q* × *f* value was mainly determined by the FWHM value and U*_b_*. A higher *Q* × *f* value corresponded to a smaller FWHM value and a larger U*_b_* value.

Table 6 presents the dielectric properties at their respective optimal sintering temperatures after substitution by different additives. As we all know, BZN ceramics have been widely studied for their excellent dielectric properties. Therefore, the improvement of dielectric properties by different additives has been widely studied. With the advancement of time and technology, the *Q* × *f* value and τ*_f_* value of BZN ceramics were optimized to some extent. Especially, the Ba_0.8_Sr_0.2_(Zn_1/3_Nb_2/3_)O_3_ ceramic with superior dielectric properties (ε_r_ = 45.25, *Q* × *f* = 72,704 GHz, and τ*_f_* = 1.26 ppm/°C) was obtained in this work. It was evident from Table 6 that ionic substitution on the B-site of BZN ceramics was not as effective as that on the A-site. The above analysis indicated that Sr^2+^ addition could well optimize the microwave dielectric properties of BZN ceramics.

## 3. Materials and Methods

In this work, Sr^2+^ doped BZN ceramics were prepared by a traditional high-temperature solid-state reaction. BaCO_3_ (99%), ZnO (99%), Nb_2_O_5_ (99.9%), and SrCO_3_ (99.7%) with high purity were weighed according to the stoichiometry of Ba_1−*x*_Sr*_x_*(Zn_1/3_Nb_2/3_)O_3_ (*x* = 0, 0.1, 0.2, 0.4, 0.6, and 0.8). Zirconia balls and deionized water were used as media, and the starting materials were placed together in a nylon tank. The nylon tank with the sample was next placed in a planetary and ball milling for 10 h. Then, the mixed solution was put into a drying oven at 85 °C to dry for 20 h. The obtained powders were put into a lithium muffle furnace to calcine from room temperature to 1200 °C at the speed of 3 °C/min, and the holding time was 4 h. The ball milling and drying process described above was repeated once. The obtained powders were then pelleted by adding 5% polyvinyl alcohol as a binder. The powders were pressed into cylindrical pieces 15 mm in diameter and 7 mm in thickness by a tableting machine. Finally, the obtained samples were sintered in the temperature range of 1425–1525 °C for 4 h at a heating rate of 3 °C/min.

The sintered ceramic samples were placed In a density balance, and their bulk density was measured by Archimedes’ water immersion principle. The theoretical density of each sample was obtained by the Rietveld refinement method using Jade. The phase constituents of BSZN ceramics powders were obtained by X-ray diffraction (XRD, miniflex600, Japan) under Cu target conditions of 50 kV and 40 mA. The test parameters were set as follows: the scanning angle range (2θ) was 10–85°, the step angle was 0.02, and the sampling time was 0.3 s per step. The XRD data of BSZN ceramics were structurally analyzed using Jade software. The relationship between the crystal structure and dielectric properties was obtained by employing Rietveld refinement. The crystal structure of BSZN ceramics was studied by Raman spectroscopy (Renishaw, Wotton-under-Edge, UK, 532 nm). The chemical composition and element valence of BSZN ceramics were investigated by X-ray photoelectron spectroscopy (VG Scientific, ESCALAB 250). The fracture surface morphology of the BSZN ceramics was presented by scanning electron microscopy (SEM, su1510, Hitachi, Japan). The porosity and average grain size (A.G.) were estimated using ImageJ software. The microwave dielectric properties of the samples were measured by the cylindrical medium resonance method. The τ*_f_* value was usually measured in a high-temperature test box at 25–85 °C. The τ*_f_* value of ceramic was generally calculated from the following equation:

(28) $$\tau_{f}=\frac{f_{85}-f_{25}}{f_{25}(85-25)}\times 10^{6}(\mathrm{ppm}/{}^{\circ}C),$$

where *f*_85_ and *f*_25_ were the resonant frequency at 85 °C and 25 °C, respectively.

## 4. Conclusions

In this work, BSZN ceramics were sintered at 1500 °C for 4 h by the traditional high-temperature solid phase method. The XRD analysis results indicated that the samples obtained in this work were all pure phases without any impurity phases. The SEM results indicated that the grain size of the sample was relatively uniform at *x* = 0.2; however, abnormal growth grains appeared with the further increase in Sr^2+^ concentration. ε_r_ first increased from 40.91 to 45.25 and then decreased to 40.55, which was correlated with the density and the ionic polarizability. Distortion of the oxygen octahedron and bond energy caused a continuous decrease in τ*_f_* value from 27.72 ppm/°C to −15.72 ppm/°C in the negative direction, obtaining a near zero τ*_f_* value of 1.26 ppm/°C at *x* = 0.2. The FWHM of A_1g_(O) mode first decreased and then increased with the increase in Sr^2+^ concentration. Consistent with the changing trend of *Q* × *f* value, the results illustrated that FWHM had a great influence on the *Q* × *f* value. As increasing *x* from 0 to 0.8, the *Q* × *f* value initially increased from 55,158 GHz to 72,704 GHz and then decreased to 53,060 GHz. The change in the *Q* × *f* value was attributed to the combined action of U*_b_* and the FWHM of A_1g_(O) mode near 788 cm^−1^. Generally, Ba_0.8_Sr_0.2_(Zn_1/3_Nb_2/3_)O_3_ ceramics sintered at 1500 °C for 4 h exhibited excellent microwave dielectric properties of ε_r_ = 45.25, *Q* × *f* = 72,704 GHz, and τ*_f_* = 1.26 ppm/°C.

## Figures and Tables

**Table 1:** Lattice parameters and unit cell volumes of Ba_1−_*_x_*Sr*_x_*(Zn_1/3_Nb_2/3_)O_3_ ceramics.

*x*	*a* = *b* = *c* (Å)	*α* = *β* = *γ* (°)	*V*_cell_ (Å^3^)
0	4.09548	90	68.65
0.1	4.08758	90	68.3
0.2	4.0796	90	67.9
0.4	4.0625	90	67.05
0.6	4.04586	90	66.23
0.8	4.02254	90	65.09

**Table 2:** EDS analysis of the BSZN samples with different Sr^2+^ concentrations.

*x*	Atomic Percentage (%)				
	Ba	Sr	Zn	Nb	O
0	26.71	/	3.56	17.59	52.14
0.1	21.35	1.97	3.75	15.91	57.02
0.2	19.76	4.95	4.13	16.78	54.38
0.4	17.04	11.21	2.88	18.05	50.82
0.6	10.75	15.88	3.47	17.99	51.91
0.8	5.3	21.36	3.42	16.36	53.56

**Table 3:** The bond ionicity (*f_i_*) and bond covalency (*f_c_*) of Ba(Zn_1/3_Nb_2/3_)O_3_ ceramics.

Bond Type	Bond Length (Å)	*f_i_* (%)	*f_c_* (%)
Ba-O	2.92	85.80	14.20
Zn-O	2.01	56.03	43.97
Nb-O	2.07	83.34	16.66

**Table 4:** Comparisons between α_theo_ and α_obs_ of Ba_1–*x*_Sr*_x_*(Zn_1/3_Nb_2/3_)O_3_ ceramics.

*x*	ε_r_	V_cell_	Z	α_theo_	α_obs_	Δ (%)
0	40.91	68.65	1	15.7567	15.243	3.26
0.1	42.88	68.30	1	15.5407	15.215	2.1
0.2	45.25	67.90	1	15.3247	15.1807	0.94
0.4	44.60	67.05	1	14.8927	14.977	0.57
0.6	42.93	66.23	1	14.4607	14.7556	2.04
0.8	40.55	65.09	1	14.0287	14.4435	2.96

**Table 5:** Bond length, octahedral distortion, bond energy, and τ*_f_* value of Ba_1−*x*_Sr*_x_*(Zn_1/3_Nb_2/3_)O_3_ ceramics.

*x*	*d*_NbO_ (Å)	*R*_NbO_	$v_{\mathrm{NbO}}$	V_NbO_	Δ_octahedral_	*E*_*b*_ (KJ/mol)	τ_*f*_ (ppm/°C)
0	2.07823 × 3	1.911	0.6364 × 3	3.9195	0.91 × 10^−4^	1060	27.72
	2.0591 × 3		0.6701 × 3				
0.1	2.05809 × 3	1.911	0.636 × 3	3.9239	1.03 × 10^−4^	1062	11.61
	2.07844 × 3		0.672 × 3				
0.2	2.06544 × 3	1.911	0.7106 × 3	3.9773	1.7 × 10^−4^	1074	1.26
	1.89159 × 3		0.6152 × 3				
0.4	2.02418 × 3	1.911	0.7347 × 3	5.3024	1.6 × 10^−3^	1034	−5.16
	1.8997 × 3		1.031 × 3				
0.6	2.04764 × 3	1.911	0.6912 × 3	5.7635	3 × 10^−3^	1016	−8.69
	1.83442 × 3		1.2299 × 3				
0.8	2.04507 × 3	1.911	0.696 × 3	6.3778	4.8 × 10^−3^	995	−15.72
	1.77869 × 3		1.4299 × 3				

**Table 6:** The microwave dielectric properties of some typical Ba(Zn_1/3_Nb_2/3_)O_3_-based ceramics.

Year	Ceramics Composition	Sintering Temperature (°C)	ε*_r_*	*Q* × *f* (GHz)	τ*_f_* (ppm/°C)	Reference
1982	Ba(Zn_1/3_Nb_2/3_)O_3_	1500	41	54,000	+30	[1]
2003	0.95Ba(Zn_1/3_Nb_2/3_)O_3_ + 0.05 BaZrO_3_	1450	42	96,000	+27	[25]
2004	Ba[(Zn_1−*x*_Co*_x_*)_1/3_Nb_2/3_]O_3_ (*x* = 0.8)	1450	33	20,248	−0.11	[26]
2010	Ba(Zn_1−*x*_Mg*_x_*)_1/3_Nb_2/3_)O_3_ (*x* = 0.4)	1400	36	94,400	+28.6	[27]
2008	Ba_1−*x*_La_2*x*/3_(Zn_0.3_Co_0.7_)_1/3_Nb_2/3_O_3_ (*x* = 0.015)	1425	34	63,159	5.21	[13]
2023	Ba_1−*x*_Sr*_x_*(Zn_1/3_Nb_2/3_)O_3_ (*x* = 0.2)	1500	45.25	72,704	1.26	This work

## Data Availability

Not applicable.

## Supplementary Materials

The following supporting information can be downloaded at: https://www.mdpi.com/article/10.3390/molecules28083451/s1, Table S1: A detailed list of the abbreviations; Figure S1: The EDS mapping and corresponding spectrum for the sample with *x* = 0; Figure S2: The EDS mapping and corresponding spectrum for the sample with *x* = 0.1; Figure S3: The EDS mapping and corresponding spectrum for the sample with *x* = 0.2; Figure S4: The EDS mapping and corresponding spectrum for the sample with *x* = 0.4; Figure S5: The EDS mapping and corresponding spectrum for the sample with *x* = 0.6; Figure S6: The EDS mapping and corresponding spectrum for the sample with *x* = 0.8.
