# Supplementary material for: Crystal Structure, Chemical Bond, and Microwave Dielectric Properties of Ba1−xSrx(Zn1/3Nb2/3)O3 Solid Solution Ceramics
Source: Molecules. 2023 Apr 13;28(8):3451. doi: 10.3390/molecules28083451 (PMC10141137; doi:10.3390/molecules28083451)
Supplement: Supplementary file 1 [file molecules-28-03451-s001.zip › molecules-2256261-supplementary.pdf]

Supplementary Information

Crystal Structure, Chemical Bond, and Microwave Dielectric Properties of $Ba_{1-x}Sr_x(Zn_{1/3}Nb_{2/3})O_3$ Solid Solution Ceramics

Lei Xiao [1], Lianwen Deng [2], Yu Zhang [1], Ping Wu [1], Wenfei Zeng [1] and Sen Peng [1,*]

1 Provincial Key Laboratory of Informational Service for Rural Area of Southwestern Hunan, Shaoyang University, Shaoyang 422000, China
2 School of Physics and Electronics, Central South University, Changsha 410083, China
* Correspondence: psen126@hnsyu.edu.cn

Results and discussion

Table S1. A detailed list of the abbreviations

Acronym	Full name explanation
BZN	$Ba(Zn_{1/3}Nb_{2/3})O_3$
BSZN	$Ba_{1-x}Sr_x(Zn_{1/3}Nb_{2/3})O_3$
XRD	X-ray diffraction
XPS	X-ray photoelectron spectroscopy
FWHM	full width at half-maximum
JCPDS	Joint Committee on Powder Diffraction Standards
DAM	defect activated mode
SEM	Scanning electron microscopy
$\Delta_{octahedral}$	octahedral distortion
AG	average grain size
ε_r	dielectric constant
$Q\times f$	quality factor
τ_f	temperature coefficient of resonant frequency
E_b	bond energy
U_b	lattice energy
SZN	$Sr(Zn_{1/3}Nb_{2/3})O_3$
A_mB_n	binary bond subtype of the multivariate crystals
f_i	bond ionicity
n	refractive index
C^μ	heteropole part
f_i^μ	ionic values of the μ-bond
f_c^μ	covalent values of the μ-bond
E_g^μ	average energy band
E_h^μ	isotropic polarization
b^μ	correction factor
$(Z_A^\mu)^*, (Z_B^\mu)^*$	the effective valence electron number
$exp(-\kappa_s^\mu r_0^\mu$	Thomas-Fermi shielding factor
d^μ	length of the u bond
m,n	number of anions and cations in A_mB_n
r_0^μ	ionic radius of the μ-bond
α_B	Bohr radius of a constant
k_F^μ	fermi wave vector
$(n_\mu)^*$	price of electronic number
v_b^μ	bond volume
N_{cA}^μ, N_{cB}^μ	coordination numbers of A and B atoms attached to μ bonds in a cell
N_b^v	bond density of μ-bonds (number of μ bonds in 1cm^3)
E_{A-A}, E_{B-B}	homonuclear bond energies
t_c	covalent co-mixing factors
t_i	ionic co-mixing factors
S_A, S_B	electronegativity of A and B ions

ΔS_B	change in electronegativity
Ei	energies contributed by the ionic bonds
Ec	energies contributed by the covalent bonds
U_{bi}^{μ}	lattice energy of the ionic part
U_{bc}^{μ}	lattice energy of covalent part
Z_{+}^{μ}	cationic valence states
Z_{-}^{μ}	anionic valence states
α_{theo}	theoretical dielectric polarizability
α_{obs}	observed dielectric polarizability
V_m	molar volume (V_{cell}/Z)
b'	constant ($4\pi/3$)
Δ	deviation
R_i	individual bond length of oxygen octahedron
$\bar{R}$	average bond length of oxygen octahedron
V_{ij}	sum of all of the valences from a given atom i
R_{ij}	bond valence parameter
d_{ij}	length of a bond between atoms i and j

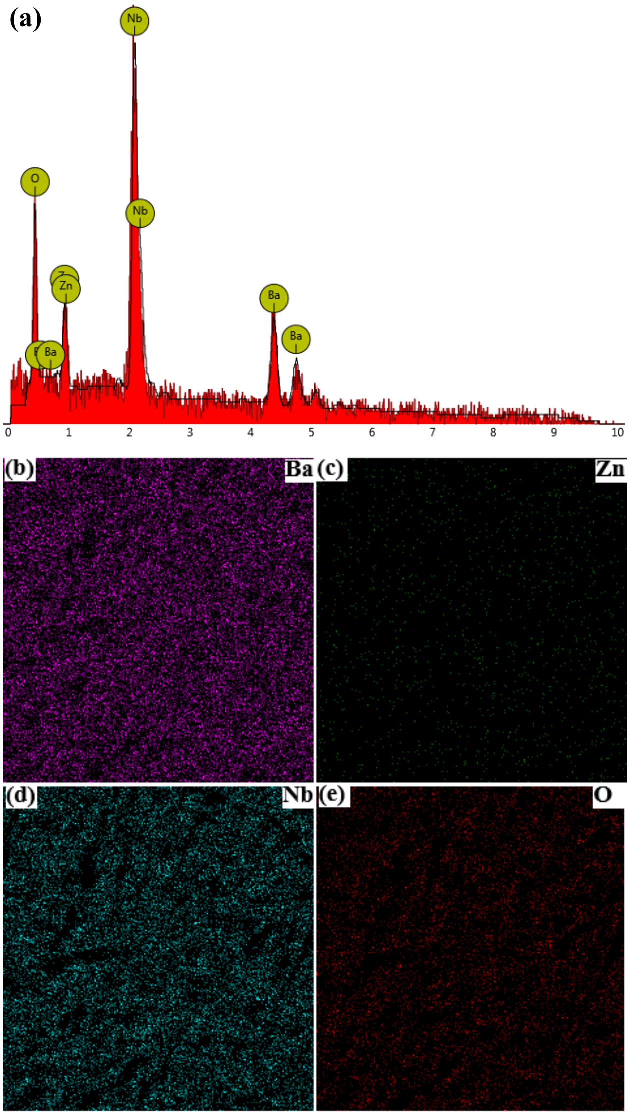

Figure S1. The EDS mapping and corresponding spectrum for the sample with $x = 0$

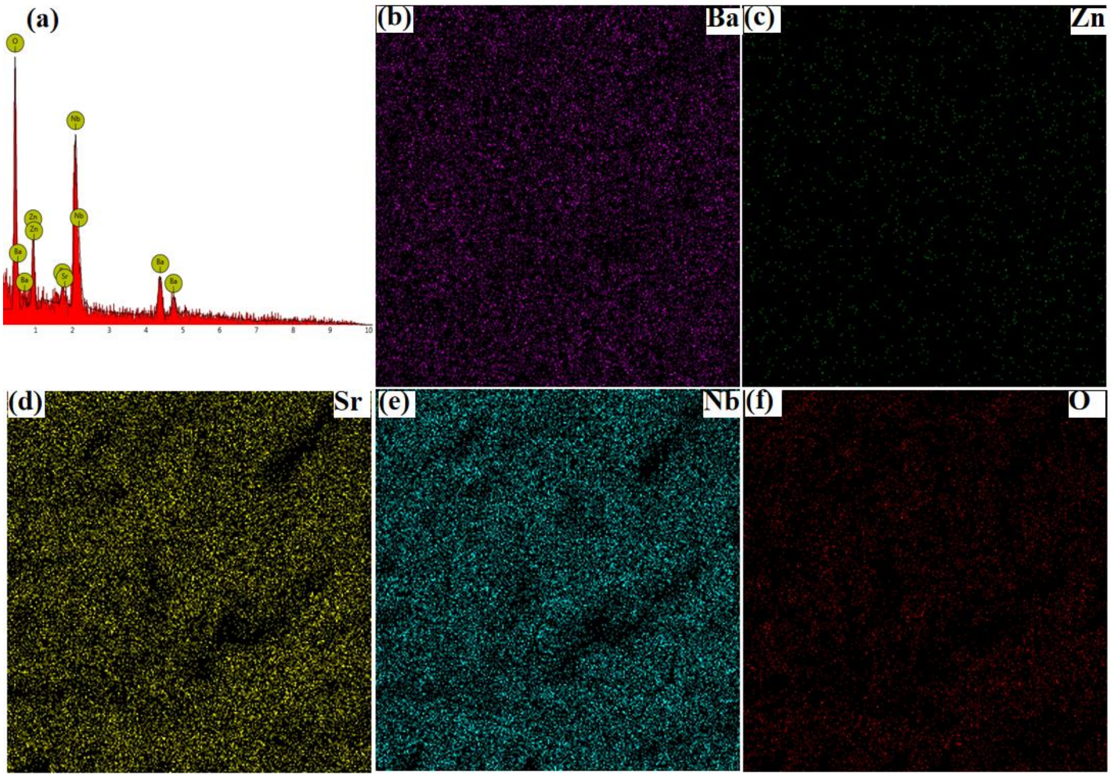

Figure S2. The EDS mapping and corresponding spectrum for the sample with $x = 0.1$

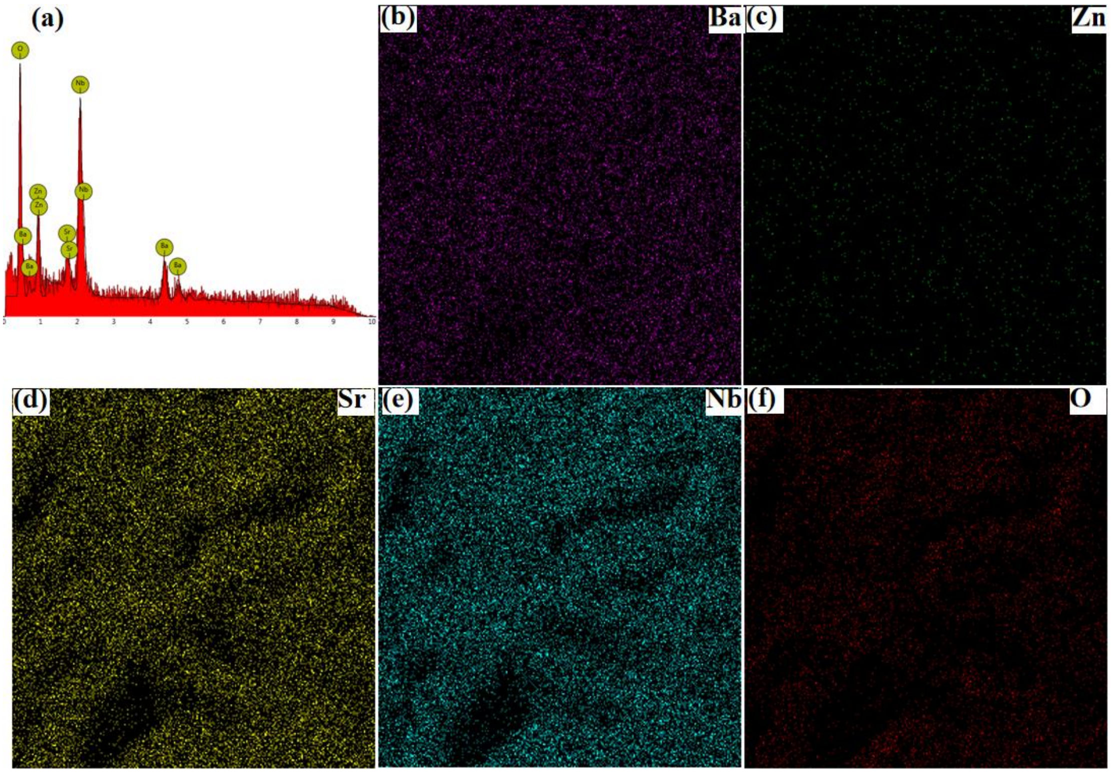

Figure S3. The EDS mapping and corresponding spectrum for the sample with $x = 0.2$

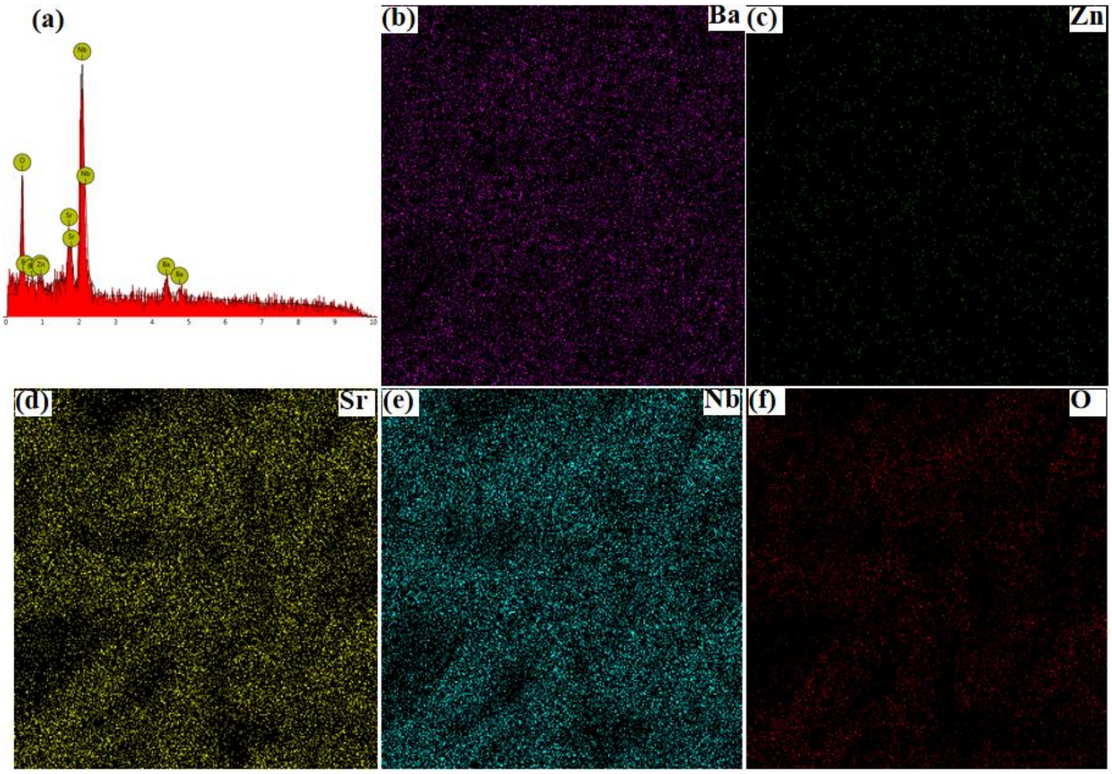

Figure S4. The EDS mapping and corresponding spectrum for the sample with x = 0.4

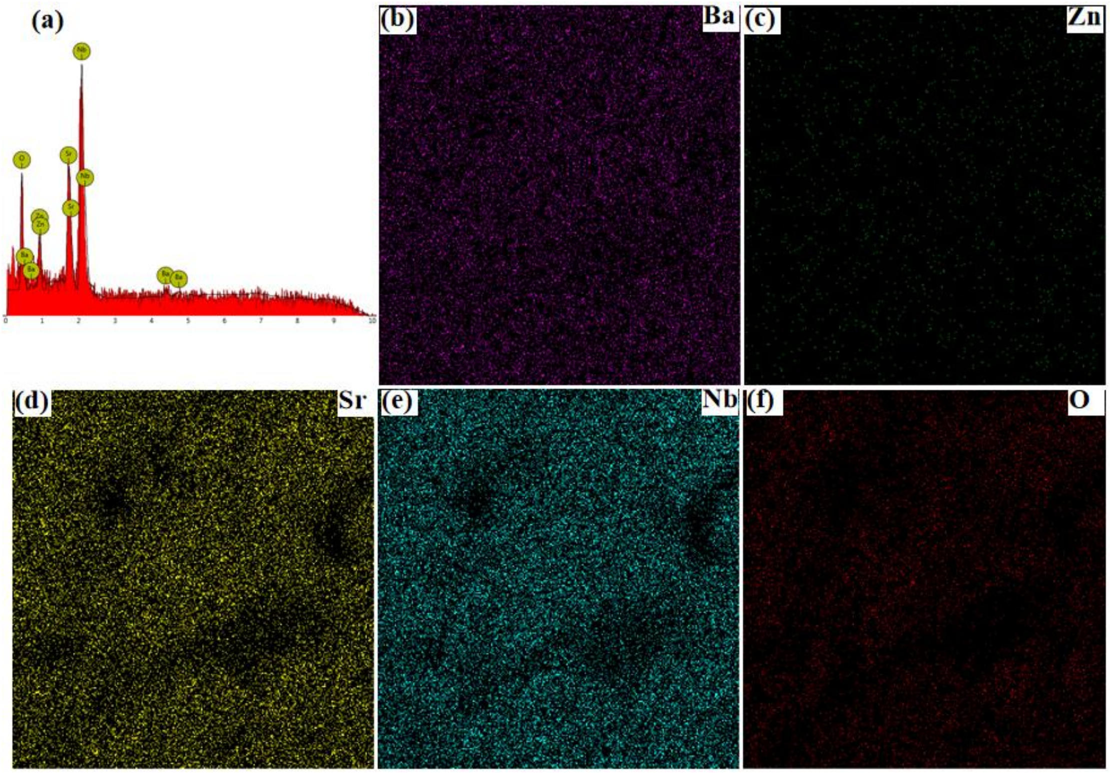

Figure S5. The EDS mapping and corresponding spectrum for the sample with x = 0.6

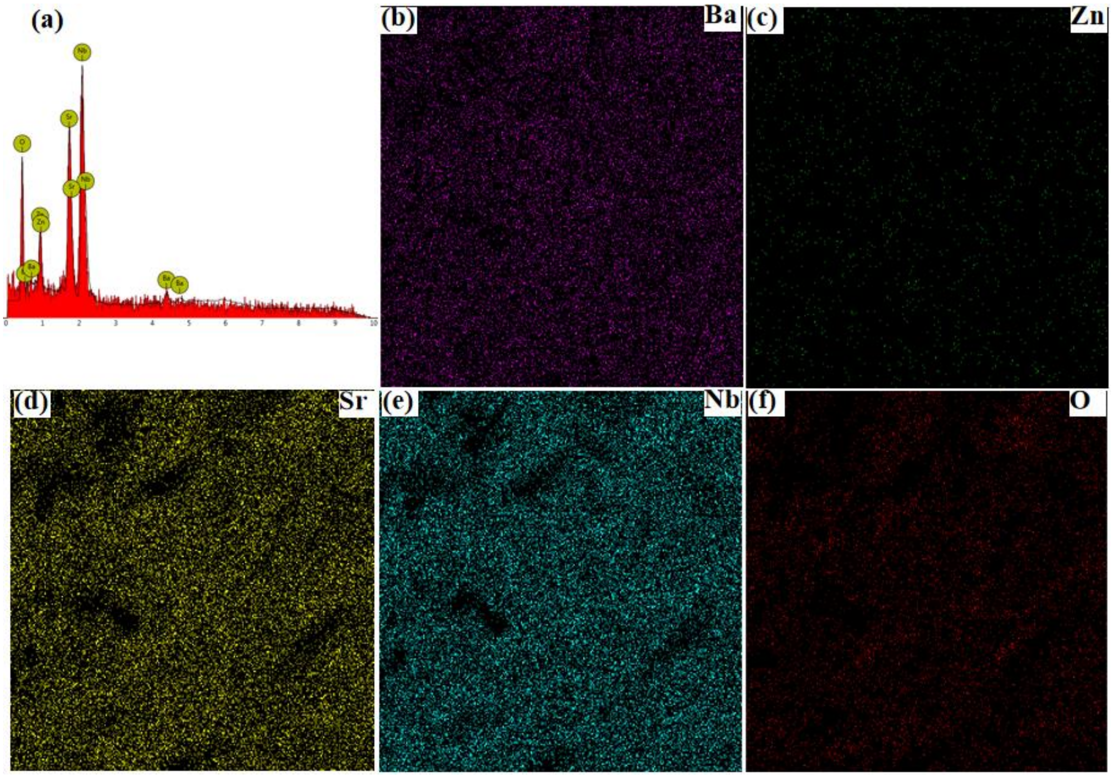

Figure S6. The EDS mapping and corresponding spectrum for the sample with x = 0.8
